# First trimester abortion protocols by facility type in Switzerland and potential barriers to accessing the service

**DOI:** 10.1038/s41598-023-34101-2

**Published:** 2023-04-26

**Authors:** Samuel Martin Eckstein, Stefanie von Felten, Laura Perotto, Romana Brun, Denise Vorburger

**Affiliations:** 1 Department of Gynaecology, Spital Limmattal, Zurich, Switzerland; 2 Department of Biostatistics at Epidemiology, Biostatistics and Prevention Institute, University of Zurich, Zurich, Switzerland; 3 Department of Gyaecology, Cantonal Hospital Winterthur, Winterthur, Switzerland; 4 Department of Obstetrics, University Hospital Zurich, Zurich, Switzerland; 5 Department of Gynaecology, University Hospital Zurich, Frauenklinikstrasse 10, 8091 Zurich, Switzerland

**Keywords:** Health services, Public health, Health care, Medical research

## Abstract

Simplified first-trimester abortion protocols are well established. However, data on the use of medical or surgical abortion protocols across Switzerland is lacking. We report protocol characteristics in abortion care for two different facility types, hospital vs private practices (office-based) in Switzerland. Furthermore, we investigate an association between protocol characteristics and the likelihood of following through with the abortion at the same facility. We also report abortion outcomes of an office-based cohort where doctors use simplified abortion protocols. This study consists of two parts. (i) Between April and July, 2019, we collected data regarding medical and surgical abortion protocols of institutions offering abortions, in a nationwide survey. We assessed whether the proportion of patients who followed through with the abortion (primary outcome) after first appointment was associated with predefined protocol characteristics, considered to complicate access to abortion services, using generalised estimating equations. (ii) We analysed abortion outcomes of six selected office-based facilities from January, 2008, to December, 2018, using simplified abortion protocols in accordance with the Worlds Health Organisation (WHO) guidelines. (i) We included a total of 39 institutions. Hospitals showed more protocol-based barriers to abortion access compared with office-based facilities. The odds of undergoing an abortion after the first appointment were increased using protocols with minimal barriers. Overall, office-based facilities applied higher gestational age limits, required fewer appointments, and administered mifepristone more often after the first visit than did hospitals. (ii) We included a total of 5274 patients with an incidence of complications requiring surgery of 2.5% in line with rates reported in published literature. Only a few hospitals provide abortion care with easy access to medical and surgical abortion, whereas most office-based facilities do. Access to abortion services is generally crucial, and should be provided in a single visit whenever clinically permissible.

## Introduction

First-trimester abortions can be performed safely either medically or surgically^1^. Since 2002, it has been a legal requirement in Switzerland for first-trimester abortions to be provided to women on demand within the first 12 weeks of pregnancy^2^. Cantonal authorities approve public hospitals as well as specialist general practitioners (GPs) and gynecologists within office-based settings to provide abortion care. Since statutory approval in 2002, the number of abortions remains stable at one of the lowest levels in Europe. Around 11,000 first-trimester abortions are carried out every year, with 6.4 per 1000 individuals aged between 15 and 49 years^3^. At present, most first-trimester abortions in Switzerland are medical, accounting for 74%, whereas 26% are conducted by suction evacuation^3^. Despite liberal abortion laws, Swiss guidelines^5^ indicate that access to service and patient privacy may be compromised for several reasons. One argument for this is that mifepristone is not approved beyond 49 days after last menstrual period (LMP)^4^, which is endorsed by the Swiss guidelines of Gynecology and Obstetrics (SGGG)^5^. Whilst the guideline considers an off-label use for later gestational ages, the responsibility lies with the patient and the service provider^5^. Another reason is that cantons impose compulsory counselling on patients seeking abortion, despite the fact that this recommendation is not mandatory according to Swiss law^2^. Moreover, home use of medical abortion medication is not common, and patients are obliged to attend another appointment in order to receive the medication. Additionally, post-abortion care following medical abortion usually requires another clinical visit comprising an ultrasound to exclude an ongoing pregnancy. Taken together, these guidelines oblige patients to attend three to four face-to-face appointments throughout the process, which delays abortion care and imposes barriers.

International guidelines generally endorse simplification of abortion care, including one-visit protocols, as this has been proven to be safe^1,6–11^. Based on clinical evidence^12–14^, these recommendations include medical abortion up to 70 days after LMP and the removal of unnecessary barriers to abortion access, such as compulsory counselling^1,9^. These guidelines also support home use of medical abortion medication and post-abortion care assessment via tele-medicine in conjunction with low-sensitivity urine pregnancy tests (LSUPC)^15–19^.

However, data on the varied use of medical or surgical abortion protocols across Switzerland by facility type is lacking. Our study intends to identify potential protocol-based barriers to abortion services with a focus on two different health-care providers: hospitals versus office-based settings. Furthermore, we investigate a comprehensive one-decade dataset obtained from six GPs in Zurich with a focus on complications requiring surgery. Their abortion protocols are straightforward and in line with the WHO guidelines. They thus support simplified access to abortion care.

## Methods

The study followed the STROBE [Strengthening the Report of observational Studies in Epidemiology] guidelines for reporting observational studies, and consists of two parts^20^.

### Part 1 nationwide survey

#### Study design and settings

We distributed a nationwide questionnaire among hospitals and private practices covering details of medical and surgical abortion protocols and numbers between April and July, 2019.

In March 2019, we developed a questionnaire comprising nineteen multiple-choice questions pertaining to medical and surgical abortion protocols (Supplementary Information, SI Table S1). This specific set of questions included answer options which are in line with the Swiss guidelines^5^, and answers which adhere to international guidelines, such as the WHO guidelines^1^. This approach allowed us to identify different practices within the abortion process. Additionally, we asked for the annual number of patients seeking abortion at each institution, as well as the annual number of abortions carried out between 2014 and 2018. As a result, we were able to identify a discrepancy between these numbers. Each questionnaire was translated into the main Swiss languages (German, French, Italian) by a native speaker with a medical background.

#### Facility recruitment and eligibility

We identified two different types of service providers offering abortion care in Switzerland. We grouped these institutions into hospitals and private practices (office-based setting). Therefore, we identified all Swiss teaching hospitals (which are listed by the Swiss Institute for Medical Education register (SIWF)^21^) as well as authorised office-based GPs (which are listed by APAC-Suisse register) which specifically offer abortion care^22^. Between April and May 2019, we contacted the directors of these institutions by post and asked them to complete the questionnaire. If a questionnaire was missing or incomplete, we recontacted the facility for further enquiry via email after 4 weeks, and again after six weeks, if necessary. The institutions were required to confirm that the questions had been answered by a fully qualified member of staff regularly involved in abortion services. In order to be eligible, the institution was required to offer both medical and surgical abortion, and all the answers on the questionnaire concerning the abortion protocol for both procedures were required to be complete.

#### Definition of protocol characteristics which complicate access to abortion care

Based on discrepancies between the Swiss and the WHO guidelines^1^ regarding abortion care, we defined protocol characteristics which may constitute barriers to accessing the service. These factors are listed in Table 1.

**Table 1:** Protocol characteristics, which may constitute barriers to accessing the service–definitions.

	Possible answers	Barriers to accessing the service
Gestational age limit—days	Up to 49/63/70/77/84 days after LMP	Lower gestational age limit in days
No. of clinical visits	1–6	Higher no. of clinical visits
Compulsory counselling and imposed time of reflection	Yes/no	Yes
Possibility of oneStopMTOP*	Yes/no	No
Possibility misoprostol home use	Yes/no	No
No. of staff members involved	1–4	More staff-members involved

*One Stop (one clinical visit) for Medical Termination of Pregnancy.

#### Outcomes

The primary outcome was the proportion of patients who followed through with the abortion at the same facility, calculated as the number of abortions carried out relative to the number of patients seeking abortion, and in each of the years 2014–2018.

#### Statistical analysis

Statistical analyses were performed in R version 4.0.4 (2021-02-15). Descriptive statistics of each facility’s characteristics and its abortion protocols were tabulated by facility type. Frequency and percentage were reported for categorical variables, and mean and standard deviation for continuous variables. We defined explanatory variables considered as protocol characteristics complicating access to abortion services (Table 1). In order to assess the association between abortion protocol characteristics and the proportion of patients who continued with their first-contact-provider, we used binomial generalised estimating equations (GEE) with facility as a cluster term due to repeated measurements per facility (2014–2018). These measurements represent the number of patients seeking abortion, and the number of abortions carried out per year and institution. We then fitted one GEE for each explanatory variable (single-variable model), and a multivariable GEE with all explanatory variables (full model). Odds ratios with 95% confidence intervals (CI) and corresponding p-values are reported.

### Part 2 patient cohort from six GPs

#### Study design and patient population

This cohort study was a retrospective analysis of a prospectively maintained and de-identified database from six office-based abortion care providers in Zurich, Switzerland. Ethical approval was obtained (BASEC-No. Req-2018-01031). We included patients with first-trimester pregnancies treated by means of medical or surgical abortion with a follow-up of at least 6 weeks between January 2008 and December 2018. Their abortion protocols are in line with the WHO guidelines^1^ and are shown in supplementary information (SI) Table S2. Follow-up after medical abortion entailed a low-sensitivity urine pregnancy test combined with a telephone call assessing patient’s symptoms and history, whereas suction evacuation follow-up included a phone call 6 weeks after surgery.

#### Outcomes

The primary outcome was the incidence of complications requiring surgery after a medical or surgical abortion, defined as suction evacuation due to incomplete abortion, ongoing pregnancy, infection, or major haemorrhage.

#### Statistical analysis

Descriptive statistics of the facility’s characteristics and their abortion protocols were tabulated. Frequency and percentage were reported for categorical variables, and mean and standard deviation for continuous variables.

#### Ethical approval

This study has been approved by the Ethical committee of the Canton of Zurich, Switzerland (BASEC-No. Req-2018-01031). We received an anonymised database from a cohort of six GPs comprising patient data which was prospectively collected and de-identified. We performed all analyses in accordance with the relevant guidelines and regulations.

### Consent for publication

Informed consent was waived by the ethics committee (Ethical committee of the Canton of Zurich, Switzerland, BASEC-No. Req-2018-01031).

### Reporting guidelines

The authors have completed the STROBE reporting checklist.

### Ethical statement

The authors are accountable for all aspects of the work, ensuring that questions related to the accuracy or integrity of any part of the work are appropriately investigated and resolved. The second part of the study contained anonymized patient data and was conducted in accordance with a clarification of responsibility, approved by the Ethics Committees of Zurich (BASEC-Nr. Req-2018-01031).

## Results

### Part 1 nationwide survey

**Figure 1:**
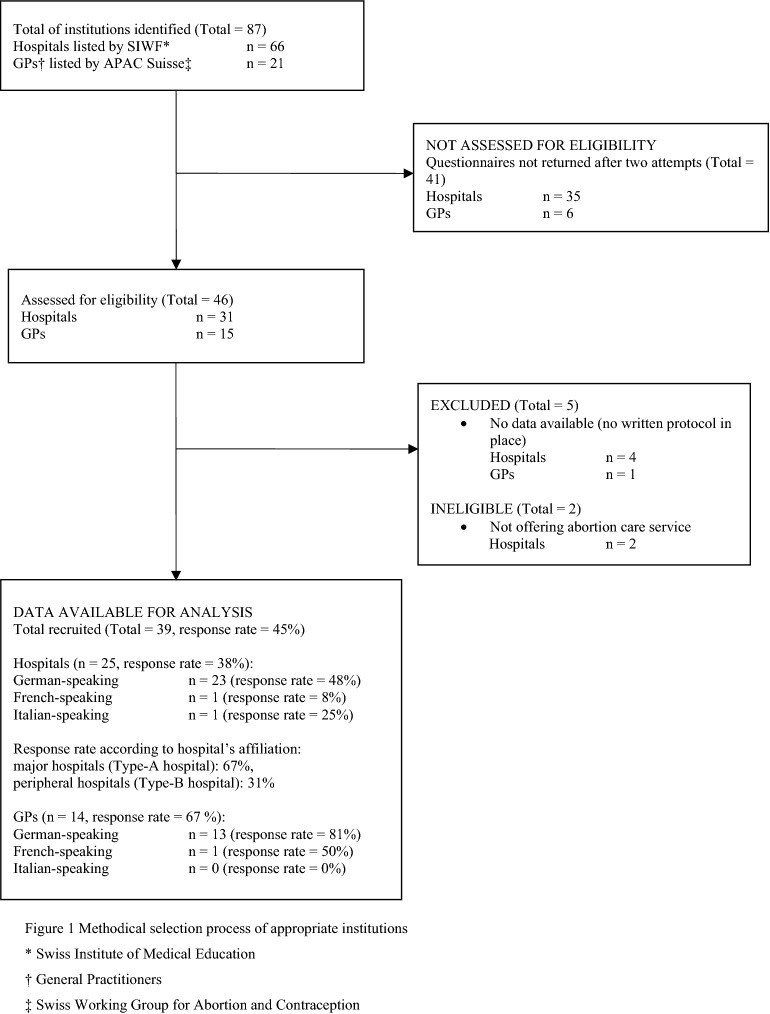
Study flow chart—Nationwide survey. Methodical selection process of appropriate institutions. *Swiss Institute of Medical Education. ^†^General Practitioners. ^‡^Swiss Working Group for Abortion and Contraception.

Searching the SIWF registry for Swiss teaching hospitals and the APAC-Suisse registry for office-based institutions (GPs) specifically offering abortion care resulted in a total of 66 hospitals and 21 office-based institutions. After two reminders, 31 hospitals and 15 GPs returned the questionnaire, corresponding to a response rate of 53%. We excluded four hospitals and one GP as they did not have a written protocol regarding abortion care. Furthermore, two hospitals were ineligible as they did not offer abortion services at all. We thus included a total of 39 institutions, 25 hospitals and 14 office-based facilities for further analysis. The study flow chart is depicted in Fig. 1. The institutions’ reported annual number of clinical visits from patients seeking medical as well as surgical abortion, and the annual number of abortions carried out between 2014 and 2018 are shown in the Supplementary Information (SI) Table S3. Additionally, the number of abortions performed in Switzerland compared with the number and proportion of abortions performed in the 39 institutions is presented in the SI Table S4.

The abortion protocol characteristics according to facility type are shown in Table 2. Office-based settings were characterised by fewer clinical visits necessitating the involvement of fewer staff members, no mandatory reflection period, a higher proportion applying a gestational age limit of more than 63 days, the availability of abortion medication for home use in the case of medical abortion, and a higher proportion of telemedicine follow-up in conjunction with a semiquantitative urine human chorionic gonadotropin test.

**Table 2:** Characteristics of abortion protocols by facility type (Hospital vs office-based). All data acquired from the questionnaires.

			Hospital n = 25	Office-based n = 14	Missing data
			n (%)	n (%)	n (%)
Gestational age limit for medical abortion (days after LMP*)—days		49	10 (40.0)	3 (21.4)	
		63	10 (40.0)	8 (57.1)	
		70	2 (8.0)	3 (21.4)	
		77	1 (4.0)	0 (0.0)	
		84	2 (8.0)	0 (0.0)	
Total			25 (100)	14 (100)	0 (0)
Total number of clinical appointments for medication abortion		1	0 (0.0)	1 (7.1)	
		2	4 (16.0)	6 (42.9)	
		3	11 (44.0)	6 (42.9)	
		4	10 (40.0)	1 (7.1)	
Total			25 (100)	14 (100)	0 (0)
Imposed time of reflection			17 (73.9)	2 (14.3)	5.1
Possibility of medical abortion induction at the first appointment			8 (34.8)	11 (78.6)	5.1
Home use after misoprostol intake			6 (25.0)	8 (57.1)	
Possibility of oneStopMToP^†^			2 (8.3)	4 (28.6)	2.6
Total number of clinical appointments for surgical abortion		2	2 (8.0)	0 (0.0)	10.3
		3	12 (48.0)	10 (100.0)	
		4	11 (44.0)	0 (0.0)	
Total			25 (100)	10 (71.4)	
Suction evacuation	Operating theatre with ward-use^⋄^		8 (32.0)	1 (10.0)	10.3
	Operating theatre without ward-use°		9 (36.0)	0 (0.0)	
	Day-care unit^∇^		8 (32.0)	1 (10.0)	
	Office-based (short stay)		0 (0.0)	8 (80.0)	
Cervical ripening with misoprostol (any gestational age)			23 (92.0)	8 (80.0)	10.3
Anaesthesia^±^	General anaesthesia		25 (100.0)	3 (30.0	10.3
	Neuraxial anaesthesia		12 (48.0)	1 (10.0)	
	Local anaesthesia		0 (0.0)	6 (60.0))	
	Local anaesthesia with sedation		0 (0.0)	7 (70.0)	
Number of staff members involved		1	8 (32.0)	11 (78.6)	0
		2	14 (56.0)	3 (21.4)	
		3	3 (12.0)	0 (0.0)	
Is a first-trimester medical abortion cost-efficient by TARMED^‡^—Yes			12 (80.0)	11 (84.6)	28.2
Is a first-trimester surgical abortion cost-efficient by TARMED^‡^—Yes			6 (40.0)	7 (77.8)	38.5
Do you think that simplified abortion protocols (e.g. oneStopMTop, home use of misoprostol and no compulsory counselling and time of reflection) decrease safety?—Yes			8 (33.3)	6 (46.2)	5.1
Do you think simplified abortion protocols increase patient comfort?—Yes			18 (75.0)	4 (33.3)	7.7
Language		G	23 (92.0)	13 (92.9)	0
		F	1 (4.0)	1 (7.1)	
		I	1 (4.0)	0 (0.0)	

*G* German, *F* French, *I* Italian; *Group 1* Hospitals, *Group 2* General Practitioners.

*Last menstrual period.

^†^One Stop (one consultation) for Medical Termination of Pregnancy.

^‡^Swiss Fee-for-Service-System.

^⋄^Patient transferred from theatres to impatient ward and discharged from there (ward staff involved).

°Patient transferred from theatres to recovery and discharged from there.

^∇^Patient transferred from theatres to day-care ward for outpatient procedures and discharged from there.

^±^Multiple answers allowed.

The vast majority of hospitals and office-based settings confirmed that the cost of a medical abortion is fully reimbursed (cost efficient) at 80% and 85%, respectively. For surgical abortion, however, hospitals were far less likely to confirm full reimbursement of costs compared with office-based institutions, at 40% and 78%, respectively.

#### Association between protocol complexity and the proportion of patients undergoing an abortion (Figs. 2, 3)

**Figure 2:**
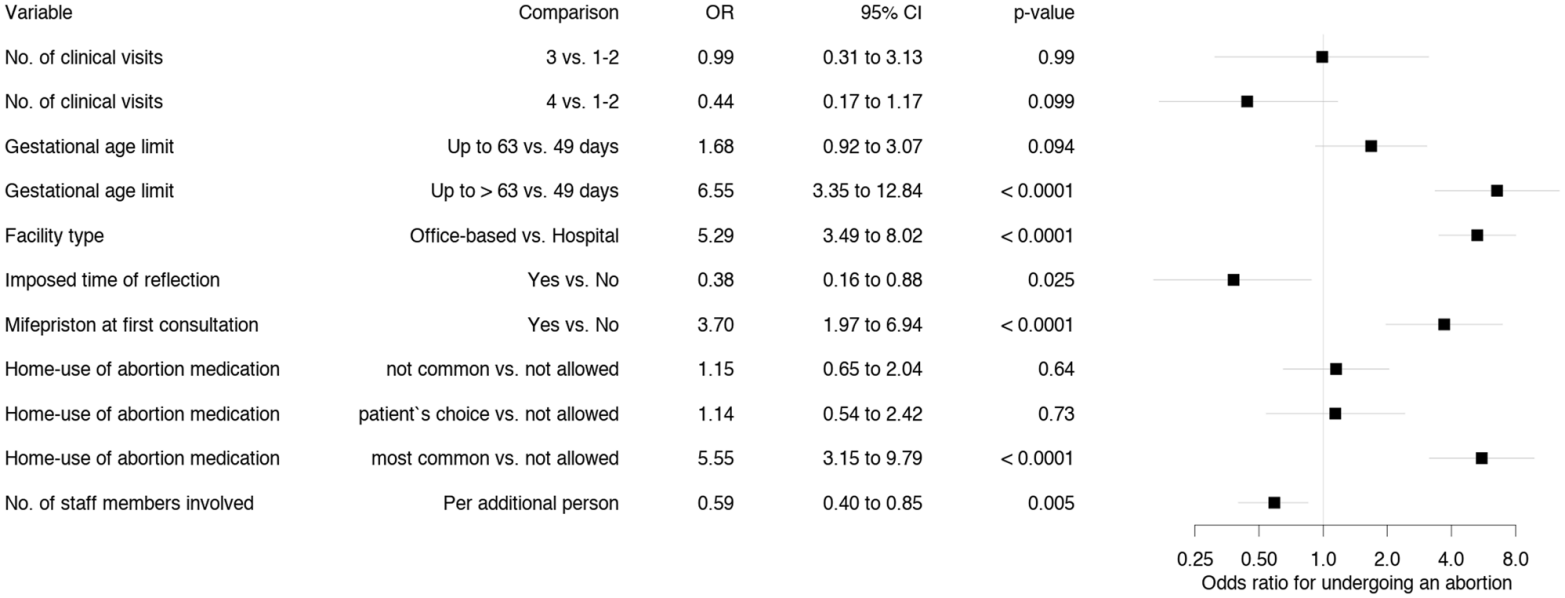
Association between protocol characteristics and the proportion of patients who followed through with the abortion at the same facility. Odds ratios were estimated by single variable GEE models (referred to as single-variable models in the text).

**Figure 3:**
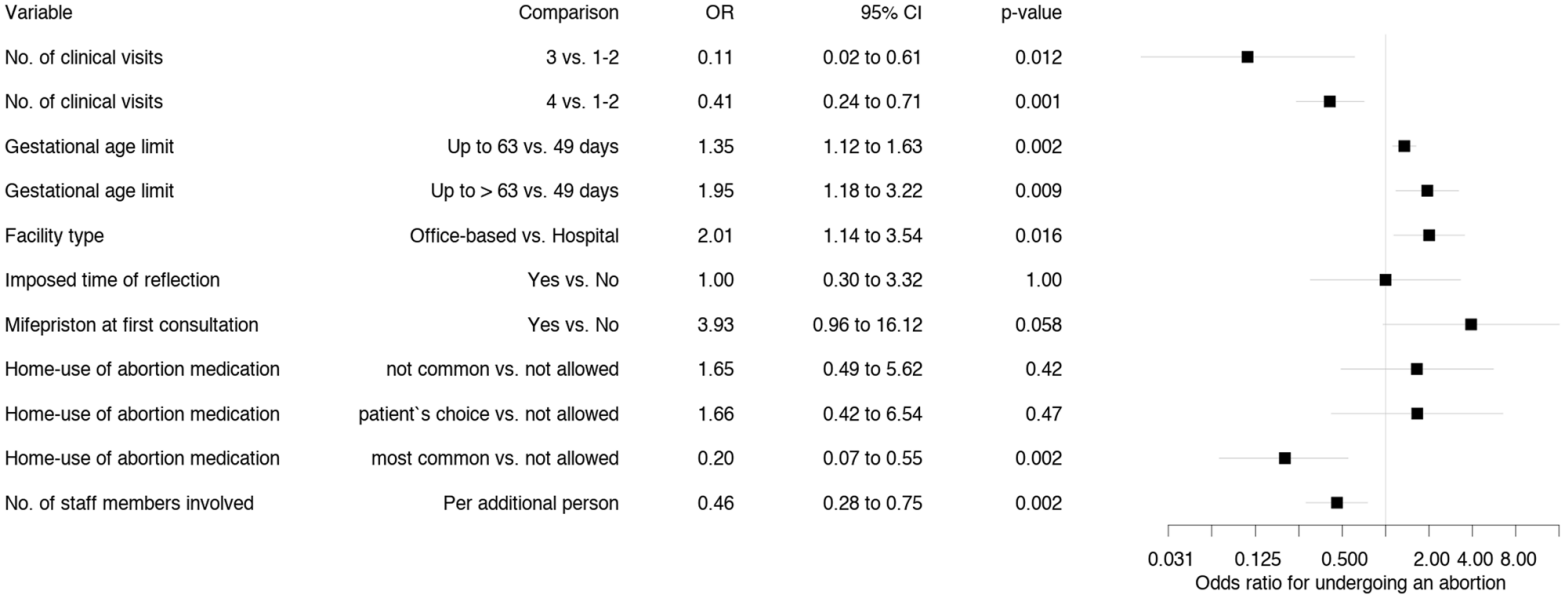
Association between protocol characteristics and the proportion of patients who followed through with the abortion at the same facility. Odds ratios were estimated by a multivariable variable GEE model (referred to as full model in the text).

Protocol characteristics complicating access to abortion services as defined in Table 1 were associated with decreased odds of following through with the abortion at the same facility after the first appointment, whereas less complex protocols led to increased odds of undergoing an abortion. Figure 2 shows the unadjusted odds ratios (ORs) from the single-variable model, whereas Fig. 3 shows the adjusted ORs from the full model.

### Part 2 patient cohort from six GPs

#### Study design and patient population

Between January 2008, and December, 2018, 5495 women sought an office-based first-trimester abortion. Patient characteristics are shown in Table 3. The proportion of medical abortions and suction evacuations changed over time: in 2009, 65% were medical abortions, and 35% were surgical. In 2018, 75% were medical and 25% surgical abortions.

**Table 3:** Characteristics of the patients treated by six general practitioners (GPs) between January 2008 and December 2018 in Zurich, Switzerland.

Characteristics		N = 5495	Missing data
		n (%)	n (%)
Age (year)—Mean (SD)		29.95 (6.81)	0 (0.0)
Age group—year	18–20	433 (7.9)	
	20–25	1086 (19.8)	
	25–30	1349 (24.5)	
	30–35	1301 (23.7)	
	35–40	929 (16.9)	
	40–45	371 (6.8)	
	> 45	26 (0.5)	
Residency	Zurich	3809 (72.8)	258 (4.7)
	All cantons in CH° except Zurich	1146 (21.9)	
	abroad	279 (5.3)	
Country of origin	CH° citizen	2604 (49.8)	264 (4.8)
	Abroad	2629 (50.2)	
No. of current pregnancy	1st	2476 (45.1)	0 (0.0)
	2nd	1252 (22.8)	
	3rd	878 (16.0)	
	4th	518 (9.4)	
	≥ 5th	371 (6.7)	
No. of previous pregnancies	0	3297 (60.0)	0 (0.0)
	1	967 (17.6)	
	2	870 (15.8)	
	3	278 (5.1)	
	≥ 4	82 (1.5)	
No. of previous first-trimester abortions	0	4214 (76.7)	0 (0.0)
	1	1027 (18.7)	
	≥ 2	254 (4.6)	
Type of abortion	Suction evacuation	1729 (31.5)	0 (0.0)
	Medical abortion	3766 (68.5)	
Gestational age (days)—Mean (SD)		49.71 (15.84)	0 (0.0)
Gestational age—days	0–56	4320 (78.6)	
	57–70	601 (10.9)	
	71–84	243 (4.4)	
	85–112	316 (5.8)	
	113–154	15 (0.3)	

*CH* Switzerland.

#### Follow-up and complication rates of patients from the six GPs

The complication rates are shown in Table 4. A total of 5,274 patients were included in the calculation of complication incidence. The mean follow-up was three months (from 6 weeks to 6 months). Two hundred and twenty-one patients (4.02%) from a total of 5495 were lost to follow-up. The incidence of complications requiring surgery was at a low level of 2.5%.

**Table 4:** Complications of the patients with abortion treatment by six general practitioners (GPs) between January 2008 and December 2018 in Zurich, Switzerland.

Complications		N = 5274	95% CI	Missing data
		n (%)	%	%
Total suction evacuation cases		133 (2.5)	2.12–2.99	0
Total of suction evacuation after medical abortion		119 (3.3)	2.76–3.96	0.8
Indication for suction evacuation after medical abortion	Major haemorrhage	24 (20.2)	13.6–28.7	0
	The patient refused repetitive misoprostol due to incomplete abortion in the absence of major haemorrhage	95 (79.8)	89.2–98.8	0
Total of reaspiration after surgical abortion		14 (0.8)	0.475–1.433	0
Indication for reaspiration after surgical abortion	Major haemorrhage	4 (28.6)	9.58–58.00	0
	Incomplete first surgical abortion needing respiration in the absence of major haemorrhage	10 (71.4)	42.0–90.4	0

## Discussion

### Part 1 nationwide survey

Taking into account discrepancies between straightforward international abortion guidelines^1,9,11^ and the Swiss guidance^4,5^, we compared nationwide first-trimester abortion protocols regarding medical and surgical abortion in hospitals as opposed to office-based settings, as the latter were hypothesised to be more straightforward and easier to access. Furthermore, we investigated associations between protocol characteristics and the proportion of patients who followed through with the abortion at the same facility.

Our results indicate that less complex protocol characteristics, which are more common among office-based facilities, are associated with higher odds of following through with the abortion at the same facility. By contrast, more complex abortion protocols, which are more common in hospitals, appear to decrease the odds that a patient will follow through with the abortion at the same facility. Office-based settings were characterised by fewer clinical visits requiring the involvement fewer staff members, no mandatory reflection period, a higher proportion applying a gestational age limit of more than 63 days, the availability of medical abortion medication for home use, and a higher proportion of telemedicine follow-up in conjunction with a semiquantitative urine human chorionic gonadotropin test.

Repeated assessments and counselling may cause delays in accessing abortion services and are not recommended by several guidelines, including those of the WHO^1,6–9,11^. Furthermore, reduced waiting times and fewer clinical visits decrease distress and improve patient experience^7,8,19,23,24^. However, an argument in favour of repeated assessments may be that more time is needed to determine the patient’s needs in terms of somatic or psychological comorbidities. Nevertheless, most women have already decided on abortion before their first contact with the care provider, and they are aware of contraceptive methods and how to use them. Hence, the imposition of a requirement for further counselling or a mandatory reflection period seems unethical and results in unnecessary barriers restricting access to this service.

Our results indicate that the gestational age limit appears to be an important protocol characteristic affecting the number of those who follow through with a medical abortion at the same facility when the service is offered up to a limit of 63 days or beyond. Hospitals were found to be more restrictive regarding the gestational age limit compared with GPs. Consequently, patients were not permitted to choose between a medical or surgical abortion and were obliged undergo a suction evacuation. This fact may have led to a decreased proportion of patients following through with an abortion at the same facility. Multiple studies have shown the safety and effectiveness of medical abortions up to 70 days of pregnancy with a regimen of 200 mg mifepristone followed by a single dose of misoprostol^1,12–14,25–27^. A systematic review conducted by Abbas et al. showed an overall success rate of 92.3% and an overall ongoing pregnancy rate of 3.1% between 64 and 70 days. No statistically significant difference in success rate could be demonstrated compared with a gestational age up to 63 days after LMP (93.9%)^14^.

Regarding home use of medical abortion medication, in the single-variable model (Fig. 2), the odds of following through with an abortion at the same facility were increased when offered (OR 5.55, 95% CI 3.15 to 9.79). In contrast, the full model (Fig. 3) suggests the opposite (OR 0.20, 95% CI 0.07 to 0.55). The reason for this discrepancy (and others) between single-variable models (which ignore all other explanatory variables) and the full model is that ORs estimated by the full model are adjusted for all other explanatory variables in the model. The full model estimates the OR for home use of medical abortion medication by patients in the same category for all other explanatory variables. Home use of medical abortion medication was permitted more often by institutions which required fewer clinical visits, and by facilities which did not impose a mandatory reflection period or which allowed for the administering of mifepristone at the first appointment. Thus, these variables partly explain the same variation, and the adjusted OR estimate for home use of medical abortion medication (independently of the other variables) is reversed. However, home use is safe and effective, showing that health professionals are no longer obliged to directly dispense the medication to patients^12,24,28–30^. Clinicians can prescribe mifepristone, misoprostol, and pain medications for home use, which may enhance patient experience and increase satisfaction and privacy, the latter of which is pivotal in abortion care. Another argument in favour of home use of abortion medication is the similarity of the situation compared with that of patients suffering from a first-trimester miscarriage, who are less prepared and who may even endure the process without relying on painkillers, despite experiencing comparable somatic and psychological pain.

In this study, only 8% of hospitals and 29% of GPs gave women the opportunity for post-abortion follow-up through self-assessment in conjunction with a semiquantitative urine human chorionic gonadotropin test instead of a routine clinical visit. In Switzerland, the distance between a patient's place of residence and an abortion provider is generally short, and insurance companies reimburse the costs for an ultrasound assessment. However, guidelines no longer endorse in-person visits with routine clinical follow-up, since remote and self-assessment along with a telephone call are viable alternatives to in-person follow-up^7,8,28^. Oppegaard et al. demonstrated that self-assessment in conjunction with a semiquantitative urine human chorionic gonadotropin test and standardised assessment of women’s symptoms were not inferior to standard clinical follow-up in terms of complication rates^15,31^. Self-assessment and telemedicine are especially valuable in resource-poor settings and sparsely populated regions, and they also proved their worth during the COVID-19 pandemic^15,32^. Experience has shown that many patients do not return to in-person follow-up after medical abortions in Switzerland, despite the fact that the costs are reimbursed. Hence, self-assessment could be an appropriate alternative, which is currently still underrepresented^15^. These arguments are supported by the fact that ongoing pregnancies are low at around 0.4–3% in early medical abortions using a standard regimen with mifepristone and misoprostol^10,16,33–35^. More importantly, patients undergoing ultrasound assessment frequently receive unnecessary interventions following a medical abortion, such as suction evacuation for misinterpreted residual abortion material caused by inexperienced providers^15^. The failure rate of surgical abortion is approximately the same as that of medical abortion, and an in-person follow-up visit is not required for the latter procedure^9^.

Cost-efficiency of surgical abortion has only been confirmed by 40% of hospitals compared to 77.8% among GPs, which might be directly related to more complicated protocols. However, amid growing concerns regarding cost reimbursement, hospitals should be encouraged to adjust their abortion protocols, as safety and effectiveness are seemingly uncompromised^10^.

The first part of the present study has several limitations. Firstly, the variables were self-reported by a representative of each institution based on questionnaires. Secondly, the response rate was low (53%), accounting for 34–41% of all abortions in Switzerland within the years 2014–2018. The French speaking part was the least represented in this study. Thirdly, following through with the abortion at the same facility is not necessarily a proxy for patient acceptability as we did not include any direct patient perspective in this study. Fourthly, we did not statistically account for repeat abortions within the same patient in the analysis plan as this appeared to be a rare event within a period of 5 years.

### Part 2 patient cohort from six GPs

Simultaneously, we aimed to analyse a considerable dataset, obtained from six specialist GPs in Zurich, in terms of complication rates requiring surgery. GPs are acknowledged for their simplified protocols. From 2008 to 2018, 5495 first-trimester abortions were performed. Of these (a total of 5495), 75% and 25% were performed by medical or surgical means, respectively. Despite simplified protocols, the overall complication rate requiring suction evacuation was at a low level of 2.5%. This rate is comparable to those of other studies investigating the same setting^10,14–16,19,36^. Major haemorrhage, ongoing pregnancies and the patient’s decision to refuse another dose of misoprostol were included in this 2.5% suction evacuation or reaspiration rate, respectively. The number lost to follow-up was low at 4.02%. There were similar complication rates compared to those reported in the literature^14^. In a retrospective cohort study conducted by Robertson et al. of more than 50,000 abortions, abortion-related morbidities and adverse events were compared by facility type (hospitals vs office-based settings). The overall proportion of patients with abortion-related morbidity or adverse events was 3.3%, which does not indicate a significant difference between the two facility types^37^.

The limitations of the second part of this study are as follows. Firstly, despite a complete dataset from six specialised GPs over a decade, it only represents a small cohort and is not nationwide. Secondly, the complication rates requiring surgery were not further specified. Retained tissue, abortion-related infection, haemorrhage, ongoing pregnancies, and missed ectopic pregnancies were not further specified. Therefore, the minor and major adverse events could not be differentiated.

## Conclusion

The study highlights the fact that few hospitals provide abortion care with easy access to medical and surgical abortion whereas most office-based facilities do. Unnecessary barriers, including repetitive counselling and undesired in-person visits, should be avoided because they are no longer recommended by international guidelines for patients seeking abortion care. Abortion services should be provided in a single visit whenever clinically permissible.

## Supplementary Information

Supplementary Tables.

## Data Availability

All data are available upon request to the corresponding author.
