# Supplementary material for: First trimester abortion protocols by facility type in Switzerland and potential barriers to accessing the service
Source: Sci Rep. 2023 Apr 26;13:6814. doi: 10.1038/s41598-023-34101-2 (PMC10131519; doi:10.1038/s41598-023-34101-2)
Supplement: Supplementary file 1 — Supplementary Tables. [file 41598_2023_34101_MOESM1_ESM.pdf]

Title page

Title

Title: First trimester abortion protocols by facility type in Switzerland and potential barriers to accessing the service

Authors and affiliations

Samuel Martin Eckstein [1], Stefanie von Felten [2], Laura Perotto [3], Romana Brun [4], Denise Vorburger [5 *]

1) Department of Gynaecology, Spital Limmattal, Zurich, Switzerland

2) Department of Biostatistic at Epidemiology, Biostatistic and Prevention Institute, University of Zurich, Switzerland

3) Department of Gyaecology, Cantonal Hospital Winterthur, Switzerland

4) Department of Obstetrics, University Hospital Zurich, Switzerland

5) Department of Gynaecology, University Hospital Zurich, Switzerland

**correspondence*

Department	Department of Gynecology
Hospital	University Hospital Zurich
Street	Frauenklinikstrasse 10
Postal code	8091
City	Zurich
Country	Switzerland
Corresponding author	Denise Vorburger
Phone	+41 76 576 57 09<br>and<br>+44 7774144553

Email	denise.vorburger@usz.ch
ORCID iD	0000-0001-5960-8414

Word count

Exclusive abstract, tables, and figures: 3147, graphic characters without space: 18605, with space: 21734

Number of figures and tables

Figures:

3

Tables:

4

Supplementary Information (SI):

4 tables

Supplementary Information (SI) **Tab S1** Questionnaire sent to all institutions regarding abortion protocols

1. General information

1.1. Please provide the following data from your medical records: Enter the annual number of first-contact clinical visits with patients seeking a surgical or medical first-trimester abortion, and provide the annual number of first-trimester abortions carried out from 2014 – 2018.

Year	2014	2015	2016	2017	2018
No. of first-trimester abortion visits					
No. of medical abortions carried out					
No. of surgical abortions carried out					
Total no. of abortions carried out					

2. Questions about medical abortion

2.1. What is the gestational age limit for a medical abortion at your facility?

 up to 49 days after the last menstrual period (LMP)
 up to 63 days after LMP
 up to 70 days after LMP
 up to 77 days after LMP
 up to 84 days after LMP
 other:

2.2. How many clinical visits do patients attend for a straightforward* abortion, from the first appointment to post abortion follow-up?
**(e.g. no ambivalence, age ≥ 16 years, no significant co-morbidities, no psychiatric illness, etc.).*

 1 2 3 4 5 6 more than 6 appointments

2.3. How long do patients wait from first contact to first appointment?

 0 1 2 3 4 5 6 7 days longer

2.4. Do you impose a time of reflection to your patients between the first appointment and mifepristone administration?
 Yes No

If the answer is yes, how many days at least?

 one day
 more than one day: ________________days

2.5. Do you allow patients the induction of a medical abortion at the first appointment (oneStopMTop=one clinical visit for medical termination of pregnancy)?

 Yes No

2.6. What is your abortion care protocol following misoprostol administration (a), and what is the route of drug intake (b)?

a. Mandatory surveillance at the institution's day care unit (about 4 hours)
 Recommended surveillance at the institution's day care unit; home-use only in exceptional cases
 Patient's choice regarding surveillance at the institution's day care unit or home-use
 Home-use of abortion medication supported by the institution

b. □ vaginal
□ oral
□ buccal
□ sublingual

2.7. What are the dosages of mifepristone and misoprostol?

mifepristone total:	□ 600 mg	□ 400 mg	□ 200 mg
misoprostol after 48 hours:	□ 400 µg	□ 600 µg	□ 800 µg

In case of a different scheme, please precise: ______________________________

2.8. Does your facility offer "OneStopMTop"† (one-stop medical termination of pregnancy)? †mifepristone intake at the initial consultation, no follow-up assessment in the facility, pregnancy self-test after two weeks at home.

□ Yes □ No

3. Questions about surgical abortion

3.1. How many consultations are generally necessary for a suction evacuation? (Initial consultation- surgical consent – surgery - follow-up)

□ 1 □ 2 □ 3 □ 4 □ 5 □ 6 □ ≥ 6

3.2. What is the average length of stay in hours after surgical abortion at your facility?

□ 3-4 □ 5-6 □ ≥ 7

3.3. How much time does it take in days between first appointment and date of surgery?

□ 0 □ 1 □ 2 □ 3 □ 4 □ 5 □ 6 □ 7 □ longer

3.4. Where is the suction evacuation performed?

□ office-based in local anesthesia without anesthesiologist
□ day-unit with anesthesiologist
□ operating theatre without postoperative ward stay
□ operating theatre with postoperative ward stay
□ other: ___________________________________

3.5. Do you recommend cervical ripening ahead of suction evacuation to all patients irrespective of gestational age?

□ Yes □ No

What is the dose of misoporostol that is used?

□ 200 µg □ 400 µg □ 600 µg □ 800 µg

How many hours before surgery is it used?

□ 1-2 □ 3-4 □ 5-6 □ ≥ 6

3.6. What kind of anaesthetic procedures are offered and usually performed? (multiple answers allowed)

□ General anaesthesia
□ Neuraxial anaesthesia
□ Local anaesthesia (intracervical-/paracervical block) with procedural sedation

□ Local anaesthesia (intracervical-/paracervical block) without procedural sedation

4. General questions concerning first-trimester abortion

4.1. How many gynaecology team members (specialist nurse, registrar, consultant) meet patients before surgery (excluding anaesthetic doctors and ward-staff)?

□ 1 person □ 2 persons □ 3 persons □ ≥ 4 persons

4.2. Is contraceptive counselling mandatory?

□ Yes □ No

4.3. Is the cost fully reimbursed to the institution providing the abortion?

For medical abortion: □ Yes □ No For surgical abortion: □ Yes □ No

4.4. Do you think simplified abortion protocols (e.g. oneStopMTop, home use of misoprostol and no compulsory counselling and time of reflection) are safe?

□ Yes □ No

Comments: ____

4.5. Do you think simplified abortion protocols lead to better patient satisfaction?

□ Yes □ No

Comments: ____

SI Table S2 Abortion protocol for medical and surgical abortion of the six general practitioners (GPs) in Zurich, Switzerland, between January 2008 and December 2018.

Gestational age limit for medical abortion (days after LMP*) - days		70
Total number of appointments for medical abortion		1 (if not required otherwise)
Imposed time of reflection		No
Possibility of medical abortion induction at the first appointment		Yes
Home-use after misoprostol intake		Yes
Possibility of oneStopMToP†		Yes
Total number of clinical appointments for surgical abortion		2
Suction evacuation	Operating theatre with ward-use◊	No
	Operating theatre without ward-use°	No
	Day-care unit∇	No
	Office-based (short stay)	Yes
Cervical ripening with misoprostol (any gestational age)		Yes
Anaesthesia	General anaesthesia	No
	Neuraxial anaesthesia	No
	Local anaesthesia	Yes
	Local anaesthesia with sedation	Yes, optional
Number of staff members involved		1

◊ Patient transferred from theatres to impatient ward and discharged from there (ward staff involved)

° Patient transferred from theatres to recovery and discharged from there

∇ Patient transferred from theatres to day-care ward for outpatient procedures and discharged from there

Supplementary Information (SI) **Tab S3** Institutions' information about the annual number of clinical visits from patients seeking abortion care, and the annual number of abortions carried out from 2014 to 2018. All data is acquired from the questionnaires.

	2014	2015	2016	2017	2018
	n (%)				
**No. of institutions**	39	39	39	39	39
**No. of first-contact appointments**	3621	3827	3598	3578	2857
No. of institutions that provided this data	18 (46)	19 (49)	19 (49)	19 (49)	20 (51)
No of institutions that did not provide this data (missing data)	21 (54)	20 (51)	20 (51)	20 (51)	19 (49)
**Total no. of abortions carried out (medical and surgical)**	3852	4112	4021	4166	3567
No. of institutions that provided this data	31 (80)	34 (87)	34 (87)	35 (90)	34 (87)
No of institutions that did not provide this data (missing data)	8 (20)	5 (13)	5 (13)	4 (10)	5 (13)
**Total no. of medical abortions**	2667	2819	2769	2872	2404
No. of institutions that provided this data	31 (80)	34 (87)	34 (87)	35 (90)	34 (87)
No of institutions that did not provide this data (missing data)	8 (20)	5 (13)	5 (13)	4 (10)	5 (13)
**Total no. of surgical abortions**	1184	1280	1250	1294	1157
No. of institutions that provided this data	31 (80)	34 (87)	34 (87)	35 (90)	34 (87)
No of institutions that did not provide this data (missing data)	8 (20)	5 (13)	5 (13)	4 (10)	5 (13)

SI Table S4 Total number of abortions in Switzerland compared to those performed within the institutions analysed from 2014 to 2018, n=39

	2014	2015	2016	2017	2018
	n (%)				
No. of abortions in Switzerland (FOPH*)	10275	10289	10273	10037	10457
No. of abortions in the 39 institutions analysed	3852 (38%)	4112 (40%)	4021 (39%)	4166 (41%)	3567 (34%)

*Federal Office of Public Health [21]
